# Supplementary material for: The interactome of intact mitochondria by cross-linking mass spectrometry provides evidence for coexisting respiratory supercomplexes
Source: Mol Cell Proteomics. 2017 Dec 8;17(2):216–32. doi: 10.1074/mcp.RA117.000470 (PMC5795388; doi:10.1074/mcp.RA117.000470)
Supplement: Supplemental Data [file supp_RA117.000470_133922_0_supp_23978_fzffwf.zip › spectra_annotation/mito_DR_spectra_annotation/4-1-1-1-96-1.pdf]

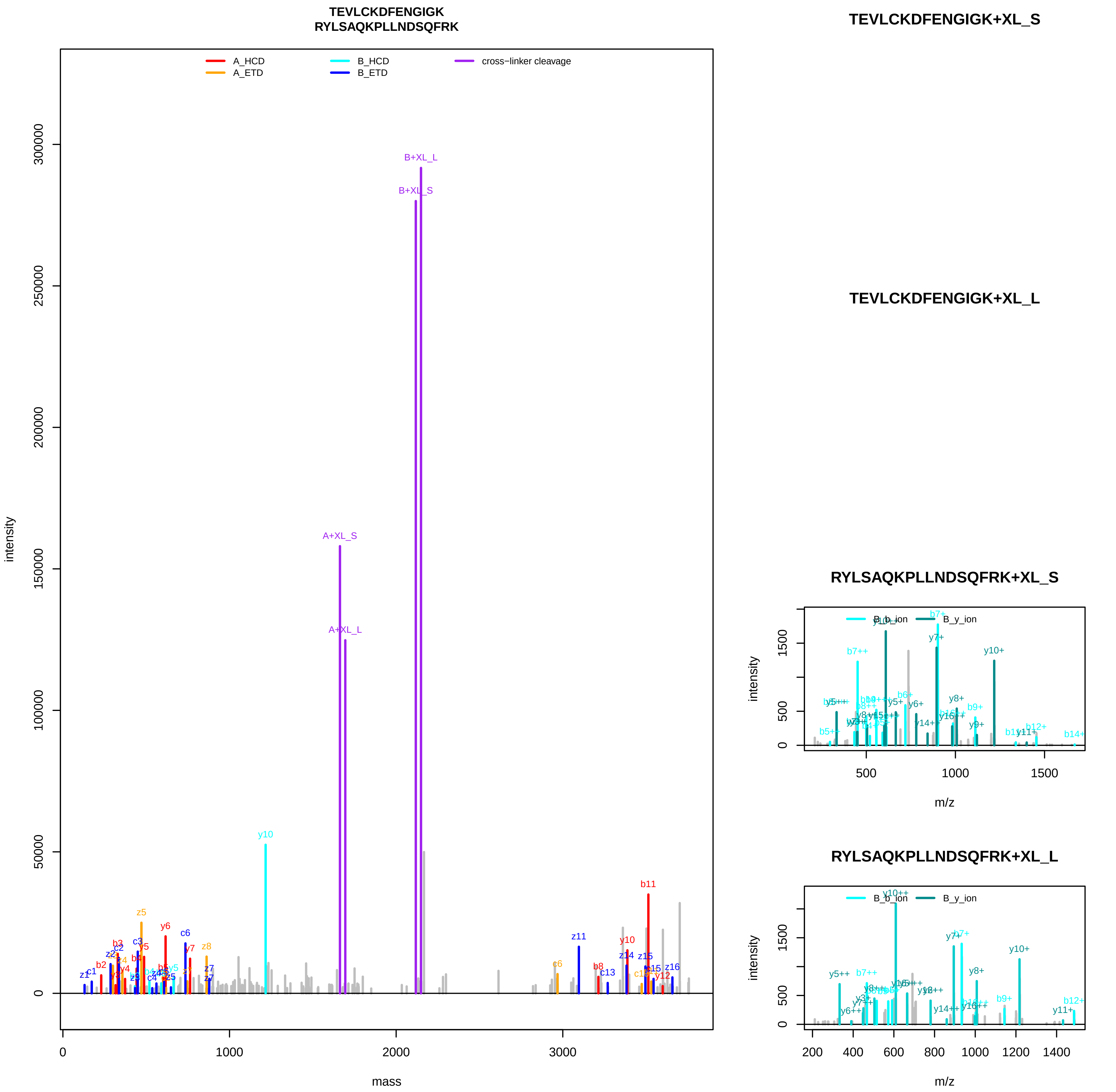

TEVLCKDFENGIGK
RYLSAQKPLLNDSQFRK
A_HCD
A_ETD
B_HCD
B_ETD
cross−linker cleavage
B+XL_L
B+XL_S
A+XL_S
A+XL_L
y10
b11
z11
y10
z5
y6
c6
z8
c6
b8
c13
z14
z15
z16
y12
intensity
mass
0
50000
100000
150000
200000
250000
300000
1000
2000
3000
TEVLCKDFENGIGK+XL_S
TEVLCKDFENGIGK+XL_L
RYLSAQKPLLNDSQFRK+XL_S
B_b_ion
B_y_ion
b7+
y7+
y10+
b7++
b6+
y6+
y8+
b9+
y9+
b12+
b14+
intensity
m/z
0
500
1500
500
1000
1500
RYLSAQKPLLNDSQFRK+XL_L
B_b_ion
B_y_ion
y10++
y7+
b7+
y10+
y8+
y5++
b7++
b9+
b12+
y11+
intensity
m/z
0
500
1500
200
400
600
800
1000
1200
1400
